# Factors Associated With Access to HIV Testing and Primary Care Among Migrants Living in Europe: Cross-Sectional Survey

**DOI:** 10.2196/publichealth.7741

**Published:** 2017-11-06

**Authors:** Ibidun Fakoya, Débora Álvarez-del Arco, Andrew J Copas, Bryan Teixeira, Koen Block, Anne-Francoise Gennotte, Alain Volny-Anne, Janneke P Bil, Giota Touloumi, Julia del Amo, Fiona M Burns

**Affiliations:** ^1^ Centre for Sexual Health and HIV Research Research Department of Infection and Population Health University College London London United Kingdom; ^2^ National Centre of Epidemiology Instituto de Salud Carlos III Madrid Spain; ^3^ Biomedical Research Network on Epidemiology and Public Health Faculty of Political Science and Sociology Universidad Complutense de Madrid Madrid Spain; ^4^ European AIDS Treatment Group Brussels Belgium; ^5^ Department of Infectious Diseases Centre Hospitalier Universitaire Saint-Pierre Brussels Belgium; ^6^ Department of Infectious Diseases Research and Prevention Public Health Service of Amsterdam Amsterdam Netherlands; ^7^ Department of Internal Medicine, Division of Infectious Diseases Center for Infection and Immunology Amsterdam, Academic Medical Center University of Amsterdam Amsterdam Netherlands; ^8^ Department of Hygiene, Epidemiology, and Medical Statistics Medical School National and Kapodistrian University of Athens Athens Greece; ^9^ Royal Free London National Health Service Foundation Trust London United Kingdom

**Keywords:** HIV, migrants, HIV serodiagnosis, primary health care, health services accessibility

## Abstract

**Background:**

There is a heavy and disproportionate burden of human immunodeficiency virus (HIV) infection among migrant communities living in Europe. Despite this, the published evidence related to HIV testing, prevention, and treatment needs for migrants is sparse.

**Objective:**

The aim of this study was to identify the factors associated with access to primary care and HIV testing among migrant groups living in Europe.

**Methods:**

A Web-based survey (available in 14 languages) was open to all people aged 18 years and older, living outside their country of birth in the World Health Organization (WHO) European area. Community organizations in 9 countries promoted the survey to migrant groups, focusing on those at a higher risk of HIV (sub-Saharan Africans, Latin Americans, gay or bisexual men, and people who inject drugs). Multivariable analysis examined factors associated with access to primary care and previous history of an HIV test.

**Results:**

In total, 559 women, 395 heterosexual men, and 674 gay or bisexual men were included in the analysis, and 68.1% (359/527) of women, 59.5% (220/371) of heterosexual men, and 89.6% (596/664) of gay or bisexual men had tested for HIV. Low perceived risk was the reason given for not testing by 62.3% (43/69) of gay or bisexual men and 83.3% (140/168) of women and heterosexual men who reported never having tested for HIV. Access to primary care was >60% in all groups. Access to primary care was strongly positively associated with living in Northern Europe compared with Southern Europe (women: adjusted odds ratio, aOR 34.56 [95% CI 11.58-101]; heterosexual men: aOR 6.93 [95% CI 2.49-19.35], and gay or bisexual men: aOR 2.53 [95% CI 1.23-5.19]), whereas those with temporary residency permits were less likely to have access to primary care (women: aOR 0.41 [95% CI 0.21-0.80] and heterosexual men: aOR 0.24 [95% CI 0.10-0.54] only). Women who had experience of forced sex (aOR 3.53 [95% CI 1.39-9.00]) or postmigration antenatal care (aOR 3.07 [95% CI 1.55-6.07]) were more likely to have tested for HIV as were heterosexual men who had access to primary care (aOR 3.13 [95% CI 1.58-6.13]) or reported “Good” health status (aOR 2.94 [95% CI 1.41-5.88]).

**Conclusions:**

Access to primary care is limited by structural determinants such as immigration and health care policy, which varies across Europe. For those migrants who can access primary care and other health services, missed opportunities for HIV testing remain a barrier to earlier testing and diagnosis for migrants in Europe. Clinicians should be aware of these potential structural barriers to HIV testing as well as low perception of HIV risk in migrant groups.

## Introduction

Within Europe, migrant populations form a substantial proportion of the number of people living with human immunodeficiency virus (HIV). From 2007 to 2012, migrants represented two-fifths of HIV cases reported and had higher odds of late presentation compared with nonmigrants [1]. In 2014, the European Centre for Disease Control and Prevention reported that 31% of new diagnoses were among people living outside their country of birth [2]. Whereas trends show that new HIV diagnoses among migrants born outside Europe are decreasing, in the 9 years to 2014, the rates of new diagnosis among European-born migrants have increased by 48% [2]. Despite the disproportionate representation of migrants in the European HIV epidemic, the published evidence base related to the HIV testing, prevention, and treatment needs is sparse and mainly focuses on sub-Saharan migrants living in the European Union [3,4].

Migrants are at an increased risk of ill health in general and sexual ill health in particular [5,6]. Studies have highlighted that cultural, financial, and structural barriers may prevent migrants from accessing HIV health care services [4,7-11]. Yet these studies focus on migrants originating from 1 region [7,9-14] or residing in 1 country [8,10-15], and few studies focus on the role of access to primary care as a gateway to providing HIV testing and earlier diagnosis [16-18]. Additionally, recent literature reviews have found that the evidence base for understanding HIV risk behaviors among migrant men who have sex with men is similarly poor [19].

This paper presents findings from the Advancing Migrant Access to Health Services in Europe (aMASE) study. This collaborative European study aimed to provide the evidence to prevent HIV infection and improve diagnosis and prognosis of migrant populations living with HIV in Europe to support policy development at the European level. Our study objectives were to identify the structural, cultural, and financial barriers to HIV prevention, diagnosis, and treatment among migrants living in Europe. We present the findings of a community participatory cross-sectional survey that identifies the factors associated with access to health care and HIV testing among multiple migrant groups.

## Methods

### Study Design

Full details of the methods used in the aMASE study have been described elsewhere [20]. In summary, all people aged 18 years and older living outside their country of birth in the World Health Organization (WHO) European area, irrespective of their HIV status or current country of residence, were eligible to participate in the aMASE community survey. The survey, hosted by FluidSurveys version 5.0 (Fluidware, now SurveyMonkey), was available online between April 2014 and July 2015.

No sampling frame for migrants in Europe exists; therefore, a convenience sampling strategy was used. In calculating the sample size, the primary outcome measures were access to primary care and the proportion of participants previously tested for HIV—both assumed to have an overall outcome prevalence of 50%. The target sample size was 1000 migrants, which allowed for estimates of the primary outcomes by gender within 5%. The survey was actively promoted from June 2014 to May 2015 using social marketing and community participatory methods in Belgium, France, Germany, Greece, Italy, The Netherlands, Portugal, Spain, and the United Kingdom. These countries were selected because they complemented data available from a companion study, the aMASE clinic survey [20]. A Community Advisory Group (CAG) comprising representatives from local community-based organizations providing services to migrant communities was contracted to promote the survey as previously described [20]. The CAG was given promotional materials, and the survey was advertised on websites, social media, and dating sites (eg, Grindr and Gay Romeo). Four population groups with unmet HIV prevention needs [21] were targeted for recruitment: sub-Saharan Africans, Latin Americans, men who have sex with men, and people who inject drugs.

The survey instrument was available in 14 languages: Amharic, Arabic, Dutch, English, French, German, Greek, Italian, Polish, Portuguese, Russian, Turkish, Spanish, and Somali. All participants provided within-survey tick-box consent.

### Ethics

Ethical approval was obtained from the London Bentham Research Ethics Committee (11/LO/1600). Additional approvals were obtained in countries that actively promoted the survey [20].

### Outcomes and Variables of Interest

The main outcome measures were access to primary care and ever having tested for HIV. Access to primary care was defined as reported possession of a health card (Italy and Spain), access to a permanent medical doctor (Greece), or registration with a family doctor or general practitioner (GP) for all other countries. Respondents who reported living with diagnosed HIV were excluded from analysis identifying factors associated with ever having tested for HIV.

Data were analyzed separately for 3 gender-related groups as it was assumed that women, men who reported their sexual orientation as straight, and men who identified as gay or bisexual were all likely to be different with regard to HIV testing history, sexual behavior, and HIV prevention needs.

Geographical variables (region of birth, region of current country of residence) were created using geographic regions and subregions classifications by the United Nations Statistics Division [22].

### Statistical Analysis

We undertook statistical analysis using Stata version 14.1 (StataCorp LLC). Individuals who reported their gender as other or who had not been—or did not intend to be—living in their current country of residence for 6 months or more were excluded from the analysis. Binary logistic regression was used to examine associations between demographic and behavior variables and the main outcome measures; we present crude odds ratios (OR) and adjusted OR (aOR) for each gender. Behavior and demographic factors that showed significant associations (alpha=.05) in a univariate analysis were included in a backwards stepwise model selection process conducted separately for each gender. We specified *a priori* that age, region of birth, sexual orientation, immigration status, and age at migration or diagnosis would be factors of interest in a multivariable analysis [20]. After models were selected, we examined whether associations between the primary outcomes and the included factors differed significantly by region of current residence or by region of birth by testing interaction terms.

## Results

The exact number of individuals invited to complete the survey or who saw advertising online or via a dating app is unknown; therefore, an overall participation rate is unavailable. It is known that 3420 individuals completed the within-survey consent form (102 or 2.98% declined to take part at that stage). Of the 3321 people who agreed to participate, 1727 went on to complete the survey, of whom 1637 (49.29% of those who consented) were included in the analysis (90 tourists were excluded from further analysis). Attrition rate analysis showed that 85.93% (2217/2580) of those who dropped out did so by question 7 (data not shown).

The 1637 respondents, of which 34.15% (559/1637) were women, reported 140 different countries of birth and 31 different countries as their current residence (9 transgender individuals were excluded from further analysis; see Multimedia Appendix 1 for full list of countries). The survey’s targeted populations were well represented, with 62.96% (673/1069) of men identifying as gay or bisexual (209/673 born in Latin America and the Caribbean), 3.96% (64/1618) of participants reporting a history of injecting drugs, and around half of women (272/559) and heterosexual men (214/396) born in Africa or Latin America and the Caribbean (Table 1).

Participants differed across gender for almost all sociodemographic characteristics, with the notable exception of age at migration (Table 1), and the average age of gay or bisexual men was lower than in the other 2 groups. Just over a fifth (148/673, 22.0%) of gay or bisexual men reported living with diagnosed HIV compared with 8.8% (49/559) of women and 6.6% (26/396) of heterosexual men (*P*<.001) (Table 1; see also Multimedia Appendices 1-3 for full demographic details).

**Table 1:** Sociodemographic characteristics of respondents, by gender (men separated by self-reported sexual orientation).

Sociodemographics		Women	Heterosexual men	Gay or bisexual men	*P* value^a^
**Current country of residence (by European region), n (%)**^b,c^					<.001
	Northern Europe	124 (22.1)	46 (11.6)	123 (18.3)	
	Southern Europe	279 (49.9)	261 (65.9)	139 (20.7)	
	Rest of Europe	156 (27.9)	89 (22.5)	411 (61.1)	
Age, years, median (IQR)^b,d^		35 (28-43)	36 (28-43)	33 (28-43)	<.001
In current country, years, median (IQR)^b,d^		8 (3-13)	8 (3-15)	5 (2-12)	<.001
Age at migration, years, mean (SD)^b,e^		26.8 (10.3)	27.2 (10.1)	26.8 (9.3)	.78
**Region of birth, n (%)**^b,f^					<.001
	Africa	155 (27.7)	167 (42.2)	36 (5.4)	
	Latin America and the Caribbean	117 (20.9)	47 (11.9)	209 (31.1)	
	Rest of the world	24 (4.3)	4 (1.0)	38 (5.7)	
	Asia	43 (7.7)	71 (17.9)	57 (8.5)	
	Europe	220 (39.4)	107 (27.2)	333 (49.5)	
**Ethnicity (n=1618), n (%)**					<.001
	Arab or Magrhebian	13 (2.4)	30 (7.7)	9 (1.3)	
	Asian	28 (5.1)	42 (10.7)	49 (7.3)	
	Black African or Caribbean	125 (22.6)	125 (31.9)	17 (2.5)	
	Latin American or Hispanic	64 (11.6)	31 (7.9)	124 (18.5)	
	Mixed ethnicity	40 (7.2)	21 (5.4)	88 (13.1)	
	Other	44 (7.9)	55 (14.0)	39 (5.8)	
	White European	201 (36.3)	83 (21.2)	302 (44.9)	
	White other	39 (7.0)	5 (1.3)	44 (6.6)	
Education: upper secondary or more, n (%)^b^		398 (71.2)	248 (62.6)	600 (89.2)	<.001
Employment: working full- or part-time, n (%)^b^		297 (53.1)	177 (44.7)	501 (74.4)	<.001
**Relationship status, n (%)**^b^					<.001
	Married or cohabitating	283 (50.6)	165 (41.7)	205 (30.5)	
	Single	206 (36.8)	182 (46.0)	397 (59.0)	
	Living apart relationship or marriage	70 (12.5)	49 (12.3)	71 (10.6)	
**Religion of those who attend services (n=627), n (%)**					<.001
	Christian (all denominations)	196 (83.4)	119 (59.5)	167 (87.0)	
	Muslim or Islam	23 (9.8)	66 (33.0)	9 (4.7)	
	Other	16 (6.8)	15 (7.5)	16 (8.3)	
**Monthly income compared with national minimum wage (n=1575), n (%)**					<.001
	More or a lot more	169 (32.1)	113 (29.4)	372 (56.0)	
	About the same	65 (12.3)	40 (10.4)	88 (13.3)	
	Less than minimum wage	122 (23.2)	89 (23.2)	120 (18.1)	
	No earnings	171 (32.5)	142 (37.0)	84 (12.7)	
	Not known	169 (32.1)	113 (29.4)	372 (56.0)	
Moderate or severe household hunger in the past 4 weeks (n=1520), n (%)		130 (25.8)	121 (34.5)	89 (13.4)	<.001
**Immigration status, n (%) (n=1625)**					<.001
	Permanent residency	343 (61.6)	183 (46.3)	482 (71.6)	
	Temporary residency	86 (15.4)	84 (21.3)	157 (23.3)	
	Asylum seeker or refugee status	35 (6.3)	63 (16.0)	9 (1.3)	
	Undocumented^f^	78 (14.0)	55 (13.9)	23 (3.4)	
	Unknown	15 (2.7)	10 (2.5)	2 (0.3)	
Living with diagnosed HIV, n (%)^b,g^		49 (8.8)	26 (6.6)	148 (22.0)	<.001
Previously injected drugs (n=1618), n (%)		19 (3.4)	20 (5.1)	25 (3.7)	.38

^a^Chi-square comparing gender-related groups.

^b^n=1628.

^c^Respondents living in Cyprus (classified as Western Asia by the United Nations but included in the World Health Organization European area) were classified as living in Southern Europe.

^d^IQR: interquartile range.

^e^SD: standard deviation.

^f^Those who checked “None of the above” in the multichoice list of all possible immigration statuses.

^g^Self-reported HIV status.

Overall, 60.8% (236/388) of heterosexual men, 73.3% (399/544) of women, and 72.6% (484/667) of gay or bisexual men reported access to a primary care physician (Table 2). The majority of participants had attended health care services in their current country of residence in the past 12 months, although 13.6% (76/559) of women, 8.8% (35/396) of heterosexual men, and 19.5% (131/673) of gay or bisexual men had returned to their country of birth and attended health care services in the previous 12 months. In each gender-related group, over 97% of the people who reported living with diagnosed HIV described visiting their HIV doctor in the last 12 months. More than a third (9/25, 36%) of heterosexual men, 25% (12/48) of women, and 4.9% (7/143) of gay or bisexual men reported missing appointments at their HIV clinic because of travel costs. Paying prescription costs was a difficulty for 53% (9/17) of heterosexual men, 41% (15/37) of women, and 19.1% (20/105) of gay or bisexual men living with diagnosed HIV.

**Table 2:** Access to health care services and human immunodeficiency virus testing among participants by gender (men separated by self-reported sexual orientation).

Services and testing variables		Women n (%)	Heterosexual men n (%)	Gay or bisexual men n (%)
Living with 1 or more chronic illnesses, not HIV^a^ (n=1628)		183 (32.7)	126 (31.8)	243 (36.1)
Has access to primary care physician (n=1599)		399 (73.3)	236 (60.8)	484 (72.6)
Visited COB^b^ for health care in the last 12 months (n=1628)		76 (13.6)	35 (8.8)	131 (19.5.)
Doctor or nurse visited in CCOR^c^ in the last 12 months (n=1625)		484 (86.6)	311 (78.9)	539 (80.2)
**Health care service attended in CCOR ever (n=1628)**				
	Any health service	519 (92.8)	361 (91.2)	587 (87.2)
	GP^d^ or family doctor	374 (66.9)	197 (49.7)	433 (64.3)
	STI^e^ clinic	75 (13.4)	48 (12.1)	346 (51.4)
	Emergency	251 (44.9)	149 (37.6)	261 (38.8)
	Outpatient	187 (33.5)	103 (26.0)	193 (28.7)
	Inpatient	148 (26.5)	99 (25.0)	153 (22.7)
	Outreach	24 (4.3)	41 (10.4)	9 (1.3)
	Antenatal	138 (24.7)	4 (1.0)	—
**HIV testing mentioned during attendance (n=1467)**				
	Any health service	196 (37.8)	129 (35.7)	374 (63.7)
	Inpatient (n=400)	31 (20.9)	26 (26.3)	28 (18.3)
	Emergency (n=661)	9 (3.6)	11 (7.4)	23 (8.8)
	Antenatal (n=142)	67 (48.6)	0 (0)	—
	STI clinic (n=469)	40 (53.3)	25 (52.1)	274 (79.2)
	Outpatient (n=483)	13 (7.0)	15 (14.6)	30 (15.5)
	Outreach (n=74)	21 (87.5)	32 (78.0)	7 (77.8)
	GP or family doctor (n=1004)	53 (14.2)	36 (18.3)	159 (36.7)
Previously tested for HIV (n=1562)		359 (68.1)	220 (59.6)	596 (89.6)
**Place diagnosed or of last test (n=1086)**				
	Antenatal service	51 (14.7)	2 (0.9)	1 (0.2)
	Hospital service (emergency, inpatient, or outpatient)	68 (19.6)	57 (26.4)	56 (10.7)
	GP or family doctor	46 (13.3)	21 (9.7)	108 (20.7)
	Sexual health clinic or HIV testing clinic	58 (16.7)	36 (16.7)	250 (47.8)
	Community or outreach setting	41 (11.8)	50 (23.1)	43 (8.2)
	Private clinic	36 (10.4)	11 (5.1)	42 (8.0)
	Other	47 (13.5)	39 (18.1)	23 (4.4)
**Reasons for never testing (n=387)**				
	Perceived low risk	140 (83.3)	125 (83.3)	43 (62.3)
	Fear of positive result and consequences	8 (4.8)	10 (6.7)	17 (24.6)
	Structural barriers to accessing health care	15 (8.9)	11 (7.3)	17 (24.6)
	Fear of test procedure	3 (1.8)	6 (4.0)	3 (4.3)
	Other	20 (11.9)	10 (6.7)	6 (8.7)
**Reasons for last test (n=1175)**				
	Perceived risk	57 (15.9)	46 (20.8)	227 (38.2)
	Feeling unwell or health problems	38 (10.6)	30 (13.6)	58 (9.7)
	Routine health checkup	148 (41.2)	96 (43.4)	337 (56.6)
	Pregnancy, blood donation, or other hospital appointment	80 (22.3)	10 (4.5)	8 (1.3)
	Job application, visa, or insurance	23 (6.4)	20 (9.0)	17 (2.9)
	Other	47 (13.1)	44 (19.9)	36 (6.1)
**People living with HIV only (n=223)**				
	Previous negative HIV test (n=208)	20 (45.5)	8 (38.1)	111 (77.6)
	Visited an HIV doctor in the past 12 months (n=221)	48 (98.0)	25 (100)	143 (97.3)
	Missed appointments because of travel costs (n=216)	12 (25.0)	9 (36.0)	7 (4.9)
	Delayed or forwent medication because of prescription costs (n=159)	15 (40.5)	9 (52.9)	20 (19.1)

^a^HIV: human immunodeficiency virus.

^b^COB: country of birth.

^c^CCOR: current country of residence.

^d^GP: general practitioner.

^e^STI: sexually transmitted infection.

The most frequently attended health service postmigration was general practice. Access to primary care (regardless of actual attendance) varied according to current region of residence (Table 3). Among women, this ranged from nearly ubiquitous access in Northern Europe (120/124, 96.8%) to less widespread access in Southern Europe at 56.4% (149/264, Table 3). This pattern was similar among heterosexual men; however, for gay or bisexual men, the lowest access to primary care was observed in the Western and rest of Europe regions (284/411, 69.1%). Multivariable analysis showed that differences in access to primary care among the regions in Europe remained significant for all 3 gender-related groups (Table 3). For women, additional factors that influenced access to care were world region of birth and immigration status. Among heterosexual men, region of birth and immigration status remain significant after adjusting for other factors, including religious practice and number of children cared for in the home (Table 3). Gay or bisexual men living with HIV were more than twice as likely to have access to primary care (aOR 2.74, 95% CI 1.53-4.86; *P*=.001), but individuals who had been resident in their current country for less than 1 year were less likely to be in receipt of primary care than those who had been in the country for 2 to 5 years (aOR 0.32, 95% CI 0.19-0.53).

**Table 3:** Factors associated with access to primary care, by gender (men separated by self-reported sexual orientation).

Factors^a^			n (%)	OR^b^	aOR^c^	95% CI	*P* value
**Women (n=541)**							
	**Current region of residence**						<.001
		Northern Europe	120 (96.8)	23.15	34.56	11.58-101	
		Southern Europe	149 (56.4)	1.00	1.00	—	
		Western and rest of Europe	130 (85.0)	4.01	5.30	3.00-9.39	
	**Age, years**						.79
		18-24	29 (67.4)	0.88	1.26	0.55-2.86	
		25-34	152 (69.4)	1.00	1.00	—	
		35-44	117 (77.0)	1.52	1.09	0.61-1.92	
		45-54	65 (75.6)	1.41	0.88	0.43-1.78	
		55+	36 (87.8)	3.27	1.78	0.59-5.32	
	**World region of birth**						<.001
		Africa	106 (72.1)	1.02	1.70	0.91-3.17	
		Latin America and the Caribbean	94 (80.3)	1.62	5.71	2.85-11.43	
		Rest of the world	45 (69.2)	0.89	0.95	0.42-2.15	
		Europe	154 (71.6)	1.00	1.00	—	
	**Years resident in CCOR**^d^						.18
		1 or less	38 (66.6)	0.49	0.65	0.29-1.46	
		2-5	91 (64.1)	0.45	0.52	0.28-0.98	
		6-9	96 (78.0)	0.89	0.97	0.51-1.82	
		10 or more	174 (79.5)	1.00	1.00	—	
	**Immigration status**						<.001
		Permanent residency	274 (80.8)	1.00	1.00	—	
		Temporary residency	51 (60.7)	0.37	0.41	0.21-0.80	
		Refugee status, unknown, or undocumented	74 (62.7)	0.39	0.64	0.33-1.23	
**Heterosexual men (n=301)**							
	**Current region of residence**						<.001
		Northern Europe	36 (83.7)	5.19	6.93	2.49-19.35	
		Southern Europe	92 (48.7)	1.00	1.00	—	
		Western and rest of Europe	53 (76.8)	2.67	2.74	1.28-5.86	
	**Age, years**						.16
		18-24	14 (48.3)	1.18	1.24	0.46-3.32	
		25-34	48 (49.0)	1.00	1.00	—	
		35-44	49 (58.3)	1.76	1.10	0.51-2.34	
		45-54	42 (73.7)	2.71	1.89	0.76-4.70	
		55+	28 (84.8)	3.72	3.94	1.14-13.57	
	**World region of birth**						<.001
		Africa	71 (61.7)	0.96	2.14	0.92-4.95	
		Latin America and the Caribbean	33 (89.2)	3.29	21.52	5.63-82.17	
		Rest of the world	24 (38.1)	0.35	1.43	0.54-3.89	
		Europe	53 (61.6)	1.00	1.00	—	
	**Years resident in CCOR**						.55
		1 or less	14 (48.3)	0.37	1.20	0.40-3.59	
		2-5	40 (46.5)	0.31	0.65	0.30-1.41	
		6-9	31 (62.0)	0.71	0.93	0.40-2.16	
		10 or more	96 (70.6)	1.00	1.00	—	
	**Immigration status**						.003
		Permanent residency	117 (77.0)	1.00	1.00	—	
		Temporary residency	28 (45.2)	0.26	0.24	0.10-0.54	
		Refugee status, unknown, or undocumented	36 (41.4)	0.29	0.42	0.18-0.97	
	**Religious practice**						.14
		Christian	84 (73.7)	1.00	1.00	—	
		Other	33 (44.6)	0.33	0.66	0.29-1.46	
		Does not attend religious services	64 (56.6)	0.50	0.51	0.25-1.05	
	**One or more child cared for in the home**						.13
		No	97 (52.4)	1.00	1.00	—	
		Yes	84 (72.4)	2.18	1.66	0.87-3.19	
**Gay or bisexual men (n=667)**							
	**Current region of residence**						.03
		Northern Europe	98 (79.7)	1.19	2.53	1.23-5.19	
		Southern Europe	102 (76.7)	1.00	1.00	—	
		Western and rest of Europe	284 (69.1)	0.68	1.28	0.74-2.20	
	**Age, years**						.38
		18-24	50 (53.8)	0.54	0.74	0.43-1.26	
		25-34	186 (68.1)	1.00	1.00	—	
		35-44	141 (78.3)	1.69	0.91	0.55-1.51	
		45-54	79 (88.8)	3.70	1.47	0.66-3.28	
		55+	28 (87.5)	3.27	2.12	0.60-7.49	
	**World region of birth**						.66
		Africa	28 (82.4)	1.75	1.83	0.63-5.28	
		Latin America and the Caribbean	152 (73.1)	1.02	1.16	0.71-1.89	
		Rest of the world	62 (67.4)	0.78	0.97	0.51-1.83	
		Europe	242 (72.7)	1.00	1.00	—	
	**Years resident in CCOR**						<.001
		1 or less	40 (38.5)	0.09	0.10	0.05-0.19	
		2-5	152 (65.5)	0.26	0.32	0.18-0.56	
		6-9	102 (88.7)	1.07	1.19	0.57-2.48	
		10 or more	190 (88.0)	1.00	1.00	—	
	**Immigration status**						.40
		Permanent residency	370 (76.9)	1.00	1.00	—	
		Temporary residency	97 (62.2)	0.49	0.86	0.51-1.45	
		Refugee status, unknown, or undocumented	17 (56.7)	0.39	0.54	0.22-1.32	
	**Living with diagnosed HIV**^e^						.001
		No	355 (68.3)	1.00	1.00	—	
		Yes	129 (87.8)	3.33	2.72	1.53-4.86	

^a^After the final model selection, each gender-related group adjusted for the factors listed under the corresponding heading in the table.

^b^OR: odds ratio.

^c^aOR: adjusted odds ratio.

^d^CCOR: current country of residence.

^e^HIV: human immunodeficiency virus.

More than two-thirds (359/527, 68.1%) of women, 59.5% (220/371) of men, and 89.6% (596/664) of gay or bisexual men had previously tested for HIV (Table 2). Respondents were asked about the place of their last test (or where they were diagnosed in the case of those living with HIV); whereas a fifth (108/523) of gay or bisexual men had tested at the GP, only 13.3% (46/347) of women and 9.7% (21/216) of heterosexual men had done so. Just over half of women and heterosexual men (40/75, 53.3%, and 25/48, 52.1%, respectively) and 79.2% (274/346) of gay men recalled being offered an HIV test in sexually transmitted infection (STI) clinics; 47.8% (250/523) of gay or bisexual men and 16.7% (58/347) of women and heterosexual men (36/216) cited an STI clinic as the place of their last test. Less than half (67/138, 48.6%) of the women who had attended antenatal care recalled being offered a test, with 14.7% (51/347) of women reporting an antenatal service as the place of their last test. Among those who had attended a GP, over a third (159/433, 36.7%) of gay or bisexual men, 14.2% (53/374) of women, and 18.3% (36/197) of heterosexual men recalled being offered an HIV test. Routine and quasi-routine health checks (such as pregnancy or other hospital appointments) were the impetus for testing for 63.5% (228/359) of women. Among all men, routine health checks and a perceived risk of HIV were the main reasons for testing.

Respondents who had never tested were asked to select their reasons for not having done so. Among women and heterosexual men, most (140/168, 83.3%, and 125/150, 83.3%, respectively) who had not tested reported that they were at no or low risk, and a few (8/168, 4.8%, and 10/150, 6.7%, respectively) reported fear of a positive test result. Although low risk of infection was a reason for not testing for 62.3% (43/69) of gay or bisexual men, around a quarter (17/16, 24.6%) also reported fears of a positive test result or structural barriers to accessing health care (Table 2).

Table 4 shows HIV risk factors among participants not living with HIV. Respondents’ basic knowledge of HIV and acquired immune deficiency syndrome (AIDS) was assessed by asking whether they “knew that AIDS was caused by a virus called HIV.” Whereas 97.1% (509/524) of gay or bisexual men responded they “knew this before today,” around 1 in 10 women (55/506, 10.9%) and 14.5% (53/366) of heterosexual men did not. A large proportion (233/500, 46.6%) of gay or bisexual men reported more than 100 lifetime sexual partners, and a similar proportion (220/520, 42.3%) reported more than 11 partners in the past year. In comparison, 2.7% (13/483) of women and 5.0% (17/338) of heterosexual men reported 100 or more sexual partners in their lifetime and the majority reported 1 or no sexual partners in the past year. A small proportion of women (20/467, 4.3%) reported no condom use in their last sexual act with a nonregular partner, and 17.2% (88/511) of gay or bisexual men reported not using a condom in their last sexual encounter with a nonregular partner.

**Table 4:** Human immunodeficiency virus (HIV) risk factors among participants not living with diagnosed HIV by gender (men separated by sexual orientation).

Risk factors		Women n (%)	Heterosexual men n (%)	Gay or bisexual men n (%)
Unaware that AIDS^a^ is caused by a virus called HIV^b^ (n=1396)		55 (10.9)	53 (14.5)	15 (2.9)
**Total number of lifetime sexual partners (n=1321)**				
	0-1	100 (20.7)	70 (20.7)	4 (0.8)
	2-5	188 (38.9)	70 (20.7)	26 (5.2)
	6-10	70 (14.5)	66 (19.5)	40 (8.0)
	11-20	64 (13.3)	56 (16.6)	44 (8.8)
	21-50	37 (7.7)	42 (12.4)	68 (13.6)
	51-100	11 (2.3)	17 (5.0)	85 (17.0)
	More than 100	13 (2.7)	17 (5.0)	233 (46.6)
**Total number of sexual partners in the past year (n=1384)**				
	None	84 (16.6)	68 (19.0)	20 (3.8)
	1	314 (61.9)	153 (42.9)	43 (8.3)
	2-10	98 (19.3)	114 (31.9)	237 (45.6)
	≥11	11 (2.2)	22 (6.2)	220 (42.3)
At last sex: no condom use (n=1308)		337 (72.2)	203 (61.5)	182 (35.6)
At last sex: no condom and nonregular partner (n=1308)		20 (4.3)	43 (13.0)	88 (17.2)
Previously diagnosed with an STI (n=952)^c,d^		74 (23.9)	44 (22.6)	227 (50.8)
Does not know where to access free condoms (n=1349)		236 (48.8)	186 (53.6)	242 (46.7)
Cannot afford condoms (n=661)^e^		167 (71.7)	112 (60.2)	201 (83.1)
**Previously exchanged sex for money, food, or drugs (n=1346)**				
	Ever	36 (7.4)	22 (6.4)	78 (15.1)
	n the last 12 months (n=126)	16 (53.3)	11 (55.0)	37 (48.7)
**Previously paid for sex (n=1561)**				
	Ever	3 (0.6)	122 (36.2)	96 (18.5)
	In the last 12 months (n=209)	0 (0)	51 (44.3)	35 (37.6)
Experienced forced sex (n=1331)		55 (11.6)	13 (3.8)	51 (10.0)
Previously used a needle to inject drugs (n=1396)		13 (2.6)	12 (3.3)	16 (3.0)
Previously shared a needle when injecting drugs (n=41)		9 (69.2)	6 (50.0)	5 (31.3)
Used drugs in the last 5 years—excluding cannabis (n=1388)		64 (12.8)	54 (14.8)	234 (44.7)
Used methamphetamine or GHB^f^ or GBL^g^ in last 5 years (n=1388)		5 (1.0)	6 (1.6)	66 (12.6)

^a^AIDS: acquired immune deficiency syndrome.

^b^HIV: human immunodeficiency virus.

^c^STI: sexually transmitted infection.

^d^Does not include those who have never tested for HIV.

^e^Only those who cannot access free condoms.

^f^GHB: gammahydroxybutyric acid.

^g^GBL: gammabutyrolactone.

In the multivariable analysis, the only HIV risk factor significantly associated with previous HIV testing in all groups was the number of lifetime partners (Table 5). For women and heterosexual men, additional associations were found for region of birth. Further factors for women were experience of forced sex (aOR 3.55, 95% CI 1.40-9.01) and receiving antenatal care postmigration (3.09, 95% CI 1.56-6.13). For heterosexual men, additional factors included access to primary care (aOR 2.67, 95% CI 1.43-4.97), and those with poorer health were less likely to have tested (aOR 0.22, 95% CI 0.22-0.84).

**Table 5:** Factors associated with ever having human immunodeficiency virus (HIV) tested among participants not living with diagnosed HIV, by gender (men separated by self-reported sexual orientation).

Factors^a^			n (%)	OR^b^	aOR^c^	95% CI	*P* value
**Women (n=426)**							
	**Current region of residence**						.13
		Northern Europe	53 (55.2)	0.62	0.51	0.26-0.99	
		Southern Europe	147 (66.5)	1.00	1.00	—	
		Western and rest of Europe	76 (69.7)	1.17	0.85	0.45-1.60	
	**Age, years**						.37
		18-24	22 (57.9)	0.61	0.55	0.23-1.30	
		25-34	119 (65.4)	1.00	1.00	—	
		35-44	81 (70.4)	1.25	1.23	0.66-2.31	
		45-54	34 (55.7)	0.62	0.74	0.32-1.67	
		55+	20 (66.7)	1.18	1.32	0.46-3.85	
	**Region of birth**						<.001
		Africa	75 (84.3)	4.00	5.42	2.48-11.83	
		Latin America and the Caribbean	66 (68.8)	1.79	2.46	1.22-4.95	
		Rest of the world	29 (55.8)	0.86	0.97	0.46-2.05	
		Europe	106 (56.1)	1.00	1.00	—	
	**Years resident in the country**						.06
		1 or less	34 (77.3)	1.31	3.09	1.13-8.44	
		2-5	60 (55.6)	0.61	0.85	0.43-1.68	
		6-9	64 (64.6)	0.86	1.01	0.52-1.95	
		10 or more	118 (67.4)	1.00	1.00	—	
	**Immigration status**						.13
		Permanent residency	187 (66.8)	1.00	1.00	—	
		Temporary residency	41 (68.3)	0.96	0.59	0.26-1.33	
		Refugee status, unknown, or undocumented	48 (55.8)	0.71	0.44	0.20-0.95	
	**Total number of lifetime sexual partners**						<.001
		0-1	37 (47.4)	0.52	0.53	0.28-1.01	
		2-5	103 (59.5)	1.00	1.00	—	
		6-10	42 (66.7)	1.28	1.54	0.76-3.11	
		More than 10	94 (83.9)	3.43	4.25	2.16-8.36	
	**Experience of forced sex**						.008
		No	235 (62.2)	1.00	1.00	—	
		Yes	41 (85.4)	3.79	3.55	1.40-9.01	
	**Children or antenatal care in CCOR**^d^						.001
		No children	127 (61.7)	1.00	1.00	—	
		Has children, no antenatal care in CCOR	69 (58.0)	0.82	0.77	0.41-1.41	
		Has children, antenatal care in CCOR	80 (79.2)	2.36	3.09	1.56-6.13	
	**Self-reported health status**						.07
		Very good	86 (78.2)	2.31	2.08	1.12-3.88	
		Good	100 (57.1)	1.00	1.00	—	
		Other response	90 (63.8)	1.30	1.39	0.78-2.46	
**Heterosexual men (n=301)**							
	**Current region of residence**						.08
		Northern Europe	20 (52.6)	1.06	0.93	0.38-2.26	
		Southern Europe	102 (51.8)	1.00	1.00	—	
		Western and rest of Europe	46 (69.7)	2.13	2.13	1.04-4.35	
	**Age, years**						.14
		18-24	22 (61.1)	1.46	1.79	0.74-4.36	
		25-34	53 (51.5)	1.00	1.00	—	
		35-44	52 (66.7)	1.65	1.86	0.90-3.81	
		45-54	26 (50.0)	0.78	0.93	0.40-2.18	
		55+	15 (46.9)	0.71	0.57	0.20-1.65	
	**Region of birth**						.01
		Africa	79 (66.4)	1.00	1.00	—	
		Latin and the Caribbean	22 (59.5)	0.54	0.38	0.16-0.93	
		Rest of the world	29 (47.5)	0.36	0.57	0.27-1.18	
		Europe	38 (45.2)	0.32	0.32	0.16-0.67	
	**Years resident in the country**						.71
		1 or less	20 (55.6)	0.85	1.45	0.54-3.95	
		2-5	51 (55.4)	1.02	1.19	0.57-2.47	
		6-9	26 (55.3)	1.08	0.80	0.35-1.85	
		10 or more	71 (56.3)	1.00	1.00	—	
	**Immigration status**						.57
		Permanent residency	81 (55.9)	1.00	1.00	—	
		Temporary residency	42 (64.6)	1.40	1.46	0.65-3.27	
		Refugee status, unknown, or undocumented	45 (49.5)	0.77	1.03	0.45-2.35	
	**Total number of lifetime sexual partners**						<.001
		0-1	23 (35.9)	0.29	0.20	0.09-0.44	
		2-5	29 (47.5)	0.47	0.39	0.19-0.80	
		6-10	35 (64.8)	0.93	1.00	0.46-2.15	
		More than 10	81 (66.4)	1.00	1.00	—	
	**Access to primary care**						<.001
		No	61 (47.3)	1.00	1.00	0.20-0.70	
		Yes	107 (62.2)	1.82	2.67	1.43-4.97	
	**Self-reported health status**						.04
		Very good	49 (55.1)	0.65	0.68	0.36-1.30	
		Good	81 (65.3)	1.00	1.00	—	
		Other response	38 (43.2)	0.40	0.43	0.22-0.84	
**Gay or bisexual men (n=492)**							
	**Current region of residence**						.64
		Northern Europe	76 (87.4)	0.97	1.18	0.53-2.61	
		Southern Europe	69 (83.1)	0.70	0.72	0.32-1.66	
		Western and rest of Europe	282 (87.6)	1.00	1.00	—	
	**Age, years**						.35
		18-24	61 (72.6)	0.34	0.53	0.26-1.09	
		25-34	188 (89.1)	1.00	1.00	—	
		35-44	115 (89.1)	1.10	0.75	0.32-1.73	
		45-54	48 (96.0)	3.27	1.86	0.36-9.62	
		55+	15 (83.3)	0.54	0.56	0.11-2.82	
	**Region of birth**						.87
		Africa	22 (81.5)	0.71	1.18	0.31-4.48	
		Latin America and the Caribbean	119 (88.1)	1.21	1.34	0.62-2.87	
		Rest of the world	69 (87.3)	1.11	1.37	0.53-3.52	
		Europe	217 (86.5)	1.00	1.00	—	
	**Years resident in the country**						.46
		1 or less	70 (76.9)	0.50	0.57	0.27-1.23	
		2-5	153 (87.9)	1.00	1.00	—	
		6-9	77 (90.6)	1.41	1.10	0.41-2.93	
		10 or more	127 (89.4)	1.17	0.81	0.34-1.94	
	**Immigration status**						.86
		Permanent residency	321 (87.5)	1.00	1.00	—	
		Temporary residency	89 (87.3)	0.94	0.99	0.44-2.21	
		Refugee status, unknown, or undocumented	17 (73.9)	0.43	0.71	0.20-2.54	
	**Total number of lifetime sexual partners**						<.001
		0-10	56 (62.9)	0.06	0.07	0.02-0.23	
		11-20	36 (78.3)	0.13	0.15	0.04-0.54	
		21-50	79 (92.9)	0.47	0.53	0.14-2.01	
		51-100	73 (91.3)	0.37	0.36	0.10-1.33	
		101-500	112 (96.6)	1.00	1.00	—	
		501 or more	71 (93.4)	0.51	0.49	0.12-1.93	

^a^After model selection, each gender-related group adjusted for the factors listed under the corresponding heading in the table.

^b^OR: odds ratio.

^c^aOR: adjusted odds ratio.

^d^CCOR: current country of residence.

## Discussion

### Principal Findings

This paper presents findings on access to primary care and HIV testing from the first European study focused on multiple migrant populations. It captures a diverse sample of migrant communities at risk of HIV infection, including migrant gay or bisexual men who form a substantial, relatively underresearched, proportion of the HIV epidemic in Europe. We have shown that determinants of access to primary care are dependent on immigration status and where an individual resides within Europe. A high proportion of participants had previously tested for HIV, but there is evidence that missed opportunities for increasing the uptake of HIV testing remain. A previous history of testing for HIV was strongly associated with sexual behavior. Accordingly, low perception of risk was identified as one of the main barriers to HIV testing among all 3 gender-related groups.

Previous studies have suggested that cultural factors act as barriers to health-seeking behavior among black African heterosexuals [10,11,17,23,24]. Whereas we found that region of origin influences access to primary care for women and heterosexual men, perhaps providing additional evidence, this association was not present for gay or bisexual men and suggests that other factors influence health-seeking behaviors in this population. Additionally, previous studies have suggested that African migrants have high rates of late diagnosis because this population has different health-seeking norms or competing priorities in comparison with Europeans [7,24]. However, this study found no significant difference in access to primary care between migrants from Africa and other regions, with the exception of Latin America and the Caribbean. This suggests that rather than sociodemographic characteristics, cultural practices, or individual health status, it is perhaps the structural factors that present the largest barriers to primary care for migrants, regardless of country of origin.

The importance of such structural or macro-level barriers may indicate changing policies and practices regarding rights to health care of both documented and undocumented migrants (including those whose visas have expired or asylum applications have been rejected). For example, in Spain, the government rescinded access to primary health care for migrants without residency papers in September 2012 only to reinstate it again in March 2015 [25,26]. In the United Kingdom, proposals to charge short-term, temporary, and undocumented migrants for primary medical care were debated in 2013 and partially implemented through a new Immigration Act in 2014 [27]. The impact of these policies regarding health care is perhaps reflected in the findings of our study. However, additional evidence is needed regarding the impact of immigration policies on access to HIV testing for migrants.

Findings are consistent with other studies showing that low perception of risk is a barrier to HIV testing for various migrant or black and minority ethnic communities [11,24,28-30]. Many of those studies focus on the needs of heterosexual migrant men and women. Our study also highlights risk perception as a barrier to testing for migrant gay or bisexual men, which may reflect a difference with nonmigrant gay men. Indeed, the European Men-Who-Have-Sex-With-Men Internet Survey Network found that HIV testing in the last 12 months was negatively associated with migrant status [31]. Given that gay or bisexual men reported fears surrounding the consequences of a positive test result and experienced structural barriers to testing (eg, having to pay for the test, not knowing where to go for a test, and being unable to test anonymously), these findings provide evidence for HIV testing initiatives aimed at migrant gay or bisexual men. Additionally, women and heterosexual men born in Africa were more likely to test than those born in other regions, but given that late presentation remains a feature for these communities [2], structural barriers may be preventing early diagnosis.

There was evidence of missed opportunities for HIV testing. Studies have shown that offering HIV tests in general practice is feasible and acceptable [17,18]. Data from this study show that general practice might be a key gateway to improving the uptake and awareness of HIV among migrant populations, particularly among heterosexual men. Given that over 80% of the participants had visited a doctor or nurse in their current country of residence in the last year and 9 out of 10 had visited some type of health service since migrating, the proportion who recalled HIV being mentioned while at a health service (47%) is comparatively low. This disparity is particularly problematic in services where there should be a policy of a routine offer or opt-out for HIV testing. Less than half of women recalled anyone mentioning HIV in antenatal care. Given that opt-out or universal HIV testing at antenatal services is available in almost all the countries featured in this survey [32], it is possible that the women tested but did not recall being offered the test. As routine testing is also available at sexual health clinics in these countries [33], the same explanation could account for our finding that nearly half of women and heterosexual men were not offered an HIV test at that service.

In addition to the structural barriers to accessing health care and HIV testing, this study provides evidence of individual-level obstacles to HIV prevention and testing. Over 1 in 10 women and heterosexual men did not have basic HIV knowledge; in all groups, around half of the respondents did not know where to access free condoms and 17% to 40% of those who did not know where to access condoms could not afford them. Whereas the finding about basic HIV knowledge might be consistent with other studies [34], we nevertheless highlight the importance of continued HIV knowledge and awareness initiatives aimed at migrant communities.

### Limitations

Our study has a number of limitations [20]. Our sample is a convenience sample; therefore, it is not representative of the European migrant population. Over half of those who consented to the survey did not complete it. Given that attrition was largely confined to the first part of the survey (the curiosity plateau), it is possible that those who dropped out at that stage had consented out of curiosity and withdrew when the survey did not meet their expectations [35]. The very large percentage of men who have sex with men in the survey reflects our targeting and advertising strategy. Individuals who fall into more than 1 population group (eg, Latin American men who have sex with men) may have been exposed to advertising and marketing on multiple occasions and different media. Nevertheless, by conducting analyses separately within gender-related subgroups, we have limited bias in our findings.

In this sample, the proportion of respondents who reported living with HIV was similar to another study that used community mobilization and engagement recruitment methods [36]. It is also possible that the high proportion of respondents who had visited an HIV doctor in the past 12 months also reflects the recruitment strategy used in this survey, particularly as around half of the respondents living with HIV reported contact with a nongovernmental organization providing HIV support (data not shown). Regardless of the recruitment methods, our findings show people living with HIV are missing appointments and forgoing or delaying medication for financial reasons. This suggests that there are possible implications for treatment adherence and service attendance.

### Conclusion

Improving access to primary care and HIV testing represents an important strategy to reduce the risk of postmigration acquisition of HIV and reduce ill health among migrants in Europe. Our data suggest that improving access to GPs, particularly for migrants without permanent residency permits, could increase the uptake of HIV testing. Clinicians and others should consider the impact of structural policies that inhibit access to HIV testing as well as interventions that increase individual knowledge, raise awareness of risk, or induce behavior change.

## Appendix

Multimedia Appendix 1 Sociodemographic characteristics of respondents.

Multimedia Appendix 2 Current country of residence of all participants in the Advancing Migrant Access to Health Services in Europe Community Survey. Color gradient indicates number of participants, with color increasing in saturation with increasing number of people. N=1637.

Multimedia Appendix 3 Country of birth of all eligible participants in the Advancing Migrant Access to Health Services in Europe Community Survey. Color gradient indicates number of participants, with color increasing in saturation with increasing number of people. N=1637.

## Abbreviations

AIDS: acquired immune deficiency syndrome
aMASE: Advancing Migrant Access to Health Services in Europe
aOR: adjusted odds ratio
CAG: Community Advisory Group
CCOR: current country of residence
COB: country of birth
FISPSE: Foundation for AIDS Research and Prevention in Spain (Spanish)
GBL: gammabutyrolactone
GHB: gammahydroxybutyric acid
GP: general practitioner
HIV: human immunodeficiency virus
IQR: interquartile range
NIHR: National Institute for Health Research
NHS: National Health Service
OR: odds ratios
STI: sexually transmitted infection
WHO: World Health Organization

## References

[1] Hernando V, Alvárez-del Arco D, Alejos B, Monge S, Amato-Gauci AJ, Noori T, Pharris A, del Amo J (2015). HIV infection in migrant populations in the European Union and European economic area in 2007-2012: an epidemic on the move. J Acquir Immune Defic Syndr.

[2] European Centre for Disease Prevention and Control, WHO Regional Office for Europe (2016). HIV/AIDS surveillance in Europe 2015.

[3] Fakoya I, Álvarez-del Arco D, Woode-Owusu M, Monge S, Rivero-Montesdeoca Y, Delpech V, Rice B, Noori T, Pharris A, Amato-Gauci AJ, del Amo J, Burns FM (2015). A systematic review of post-migration acquisition of HIV among migrants from countries with generalised HIV epidemics living in Europe: implications for effectively managing HIV prevention programmes and policy. BMC Public Health.

[4] Weine SM, Kashuba AB (2012). Labor migration and HIV risk: a systematic review of the literature. AIDS Behav.

[5] Haour-Knipe M (2000). Migration and HIV/AIDS in Europe. AIDS Infotheque.

[6] Rechel B, Mladovsky P, Ingleby D, Mackenbach JP, McKee M (2013). Migration and health in an increasingly diverse Europe. Lancet.

[7] Prost A, Elford J, Imrie J, Petticrew M, Hart GJ (2008). Social, behavioural, and intervention research among people of Sub-Saharan African origin living with HIV in the UK and Europe: literature review and recommendations for intervention. AIDS Behav.

[8] Burns FM, Johnson AM, Nazroo J, Ainsworth J, Anderson J, Fakoya A, Fakoya I, Hughes A, Jungmann E, Sadiq ST, Sullivan AK, Fenton KA, SONHIA Collaboration Group (2008). Missed opportunities for earlier HIV diagnosis within primary and secondary healthcare settings in the UK. AIDS.

[9] Fakoya I, Reynolds R, Caswell G, Shiripinda I (2008). Barriers to HIV testing for migrant black Africans in Western Europe. HIV Med.

[10] Manirankunda L, Loos J, Debackaere P, Nöstlinger C (2012). “It is not easy”: challenges for provider-initiated HIV testing and counseling in Flanders, Belgium. AIDS Educ Prev.

[11] Manirankunda L, Loos J, Alou TA, Colebunders R, Nöstlinger C (2009). “It's better not to know”: perceived barriers to HIV voluntary counseling and testing among sub-Saharan African migrants in Belgium. AIDS Educ Prev.

[12] Evans AR, Parutis V, Hart G, Mercer CH, Gerry C, Mole R, French RS, Imrie J, Burns F (2009). The sexual attitudes and lifestyles of London's Eastern Europeans (SALLEE Project): design and methods. BMC Public Health.

[13] Weine S, Bahromov M, Loue S, Owens L (2012). Trauma exposure, PTSD, and HIV sexual risk behaviors among labor migrants from Tajikistan. AIDS Behav.

[14] Wirtz AL, Zelaya CE, Peryshkina A, Latkin C, Mogilnyi V, Galai N, Dyakonov K, Beyrer C (2014). Social and structural risks for HIV among migrant and immigrant men who have sex with men in Moscow, Russia: implications for prevention. AIDS Care.

[15] Angel C, Paz Bermúdez M (2011). Native and immigrant adolescents in Spain: adaptation and perceived discrimination as HIV-risk factors. Int J Clin Health Psychol.

[16] Esteban-Vasallo MD, Morán-Arribas M, García-Riolobos C, Domínguez-Berjón MF, Rico-Bermejo J, Collado-González S, Jiménez-García R, Guionnet A, de la Fuente BP, El Kertat R, Coundoul A, Martín-Gil RA (2014). Targeted rapid HIV testing in public primary care services in Madrid. Are we reaching the vulnerable populations?. Int J Infect Dis.

[17] Loos J, Manirankunda L, Hendrickx K, Remmen R, Nöstlinger C (2014). HIV testing in primary care: feasibility and acceptability of provider initiated HIV testing and counseling for sub-Saharan African migrants. AIDS Educ Prev.

[18] Prost A, Griffiths CJ, Anderson J, Wight D, Hart GJ (2009). Feasibility and acceptability of offering rapid HIV tests to patients registering with primary care in London (UK): a pilot study. Sex Transm Infect.

[19] Lewis NM, Wilson K (2017). HIV risk behaviours among immigrant and ethnic minority gay and bisexual men in North America and Europe: a systematic review. Soc Sci Med.

[20] Fakoya I, Álvarez-Del Arco D, Monge S, Copas AJ, Gennotte AF, Volny-Anne A, Göpel S, Touloumi G, Prins M, Barros H, Staehelin C, Del Amo J, Burns FM (2016). Advancing migrant access to health services in Europe (AMASE): protocol for a cross-sectional study. JMIR Res Protoc.

[21] del Amo J, Pérez-Cachafeiro S, Hernando V, González C, Jarrín I, Bolúmar F (2010). Migrant health: epidemiology of HIV and AIDS in migrant communities and ethnic minorities in EU/EEA countries.

[22] (2017). United Nations Statistics Division: Methodology.

[23] Burns F, Fenton KA (2006). Access to HIV care among migrant Africans in Britain. What are the issues?. Psychol Health Med.

[24] Burns FM, Imrie JY, Nazroo J, Johnson AM, Fenton KA (2007). Why the(y) wait? Key informant understandings of factors contributing to late presentation and poor utilization of HIV health and social care services by African migrants in Britain. AIDS Care.

[25] (2015). Illegal immigrants to regain free healthcare.

[26] Tremlett G (2012). Immigrants in Spain to lose right to public healthcare.

[27] Gower M (2015). Immigration Health Surcharge: common casework questions.

[28] Burns F, Fenton KA, Morison L, Mercer C, Erens B, Field J, Copas AJ, Wellings K, Johnson AM (2005). Factors associated with HIV testing among black Africans in Britain. Sex Transm Infect.

[29] Anderson J (2008). Coming and going: some aspects of care for migrants with HIV in the UK. J Infect.

[30] Anderson M, Elam G, Gerver S, Solarin I, Fenton K, Easterbrook P (2010). “It took a piece of me”: initial responses to a positive HIV diagnosis by Caribbean people in the UK. AIDS Care.

[31] (2013). EMIS 2010: The European Men-Who-Have-Sex-With-Men Internet Survey. Findings from 38 countries.

[32] Savolainen-Kopra C, Kontio M, Lindeman J, Isojärvi J, Liitsola K, Mäkelä M, ECDC (2016). Antenatal screening for HIV, hepatitis B, syphilis and rubella susceptibility in the EU/EEA—addressing the vulnerable populations.

[33] Mounier-Jack S, Nielsen S, Coker RJ (2008). HIV testing strategies across European countries. HIV Med.

[34] Hickson F, Owuor J, Weatherburn P, Reid D, Hammond G, Jessup K (2009). Bass Line 2008-09: assessing the sexual HIV prevention needs of African people in England. Project Report.

[35] Eysenbach G (2005). The law of attrition. J Med Internet Res.

[36] Sadler KE, McGarrigle CA, Elam G, Ssanyu-Sseruma W, Davidson O, Nichols T, Mercey D, Parry JV, Fenton KA (2007). Sexual behaviour and HIV infection in black-Africans in England: results from the Mayisha II survey of sexual attitudes and lifestyles. Sex Transm Infect.

