# Supplementary figures and images for: Factors Associated With Access to HIV Testing and Primary Care Among Migrants Living in Europe: Cross-Sectional Survey
Source: JMIR Public Health Surveill. 2017 Nov 6;3(4):e84. doi: 10.2196/publichealth.7741 (PMC5696579; doi:10.2196/publichealth.7741)

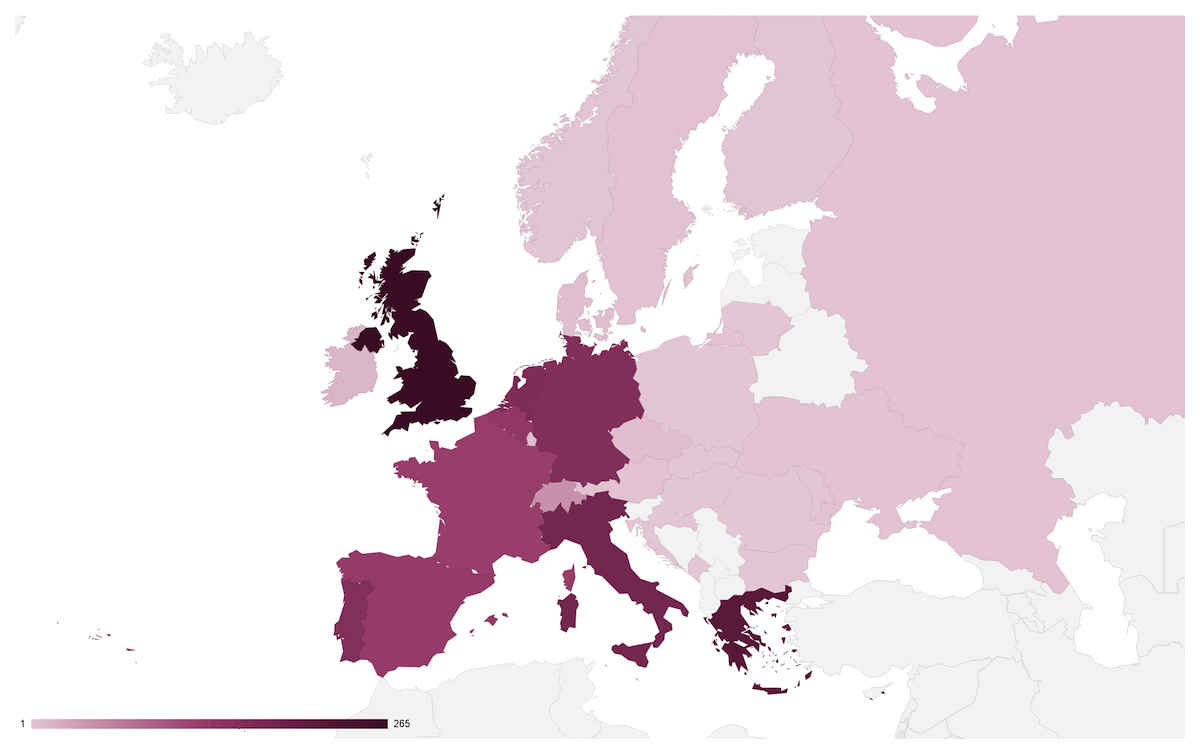

Supplement: Multimedia Appendix 2 [file publichealth_v3i4e84_app2.jpg]

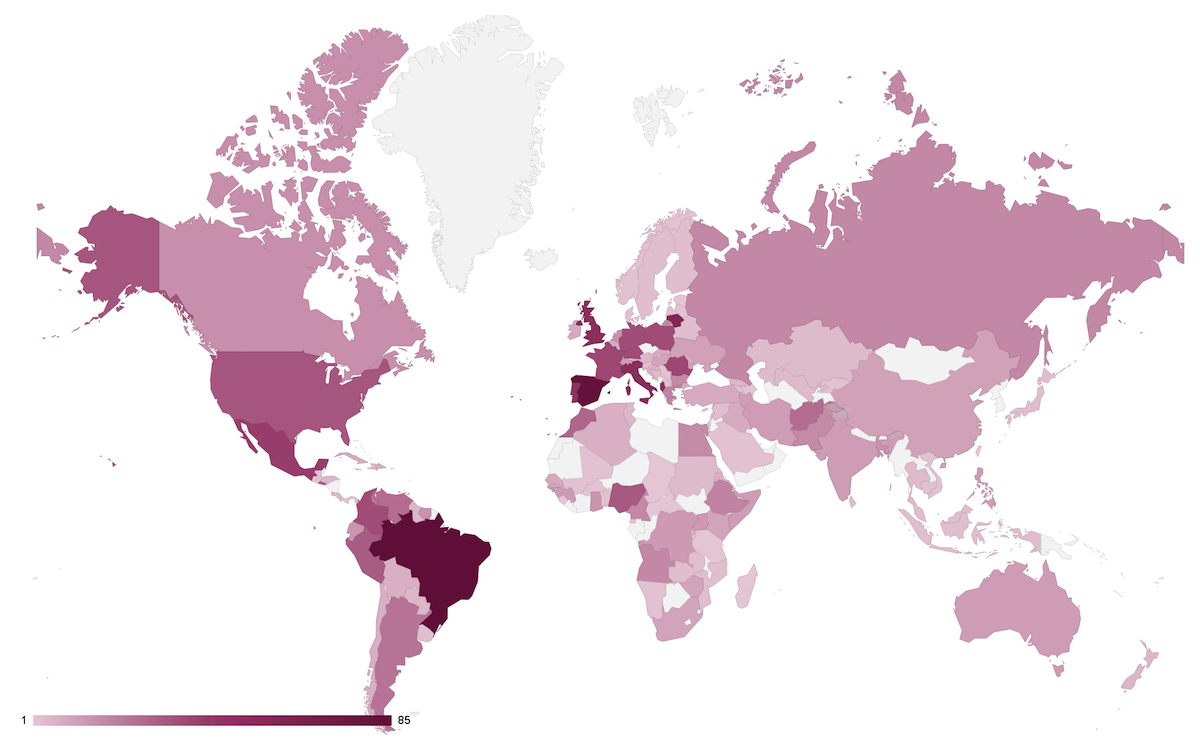

Supplement: Multimedia Appendix 3 [file publichealth_v3i4e84_app3.jpg]
